# Circulating miR-1260a as a potential biomarker for First-Episode of Psychosis: a replication study in a treatment-Naïve Romanian cohort

**DOI:** 10.3389/fpsyt.2026.1947759

**Published:** 2026-10-02

**Authors:** Ștefania-Alexandra Grosu, Maria Dobre, Iulia Andreea Pelisenco, Radu-Mihai Paun, Elena Milanesi, Mihail Eugen Hinescu

**Affiliations:** 1 Faculty of Medicine, Carol Davila University of Medicine and Pharmacy, Bucharest, Romania; 2 “Professor Doctor Alexandru Obregia” Psychiatric Clinical Hospital, Bucharest, Romania; 3 Victor Babes, National Institute of Pathology, Bucharest, Romania; 4 Aproape Clinic, Bucharest, Romania

**Keywords:** adolescents’, first-episode psychosis (FEP), microRNAs, peripheral biomarkers, plasma, schizophrenia spectrum disorders, treatment-naïve

## Abstract

The diagnosis of first-episode psychosis (FEP) in adolescents and young adults remains primarily clinical, often complicated by symptom overlap and the heterogeneity of psychiatric presentations. There is a critical need for non-invasive, objective biomarkers to support early diagnosis and patient stratification. This study aimed to replicate a panel of 10 candidate microRNAs (miRNAs) identified in a previous exploratory study within a larger, independent cohort of treatment-naïve Romanian adolescents and young adults. This cross-sectional study included 50 participants (22 drug-naïve FEP patients and 28 age- and sex-matched controls). Total RNA was isolated from plasma, and the levels of miRNAs were measured using qRT-PCR. Clinical symptoms were assessed using standardized psychiatric scales (PANSS, HDRS, YMRS). We successfully replicated miR-1260a as significantly modified in the plasma of treatment-naïve FEP patients compared to controls (FDR Adj. p = 0.04). ROC analysis for miR-1260a yielded an AUC of 0.741 (p = 0.004). Furthermore, exploratory correlation analyses conducted exclusively within the FEP patient group indicated a negative correlation between miR-1260a levels and the positive PANSS score (p = 0.017, rho = -0.503), which did not retain statistical significance after FDR correction (FDR Adj. p = 0.102). Our results corroborate and enhance our prior exploratory research, highlighting miR-1260a as a prominent candidate among circulating biomarkers for early psychosis. Future longitudinal studies are necessary to evaluate the diagnostic, prognostic, and clinical monitoring efficacy of this miRNA.

## Introduction

Schizophrenia spectrum disorders are among the most disabling psychiatric conditions, causing disability in social, occupational, and educational functioning, as well as reduced quality of life and increased healthcare burden worldwide (1). Psychotic disorders are a category of severe mental illnesses characterized by disturbances in perception, thought, cognition, emotional processing, and behavior. The formal diagnosis is made based on the presence of positive symptoms, such as delusions or hallucinations, negative symptoms, such as affective flattening, alogia, or avolition, disorganized speech, behavior, or catatonia (2).

The onset of psychotic disorders occurs predominantly during adolescence and early adulthood, which coincides with a period of neurodevelopmental changes involving synaptic pruning, cortical maturation, and reorganization of neural connectivity (3). Epidemiological data indicate that schizophrenia spectrum disorders typically emerge in early adulthood, with a median age at onset of approximately 25 years and a peak incidence around 20 years of age. Importantly, nearly half of all cases are diagnosed before the age of 25. Males generally experience an earlier onset than females and tend to exhibit a less favorable clinical course, characterized by higher relapse rates and lower remission rates (4). Similar to most mental disorders, the current diagnosis of schizophrenia and other psychotic disorders relies entirely on clinical evaluation and symptom-based classification systems, such as the DSM (2) or the ICD (5). This approach comes with several limitations, such as the symptoms being nonspecific, fluctuating, or overlapping with affective or developmental disorders, especially in the prodromal and early stages of illness (6).

Establishing a diagnosis may be especially challenging in youth, due to the heterogeneity of the clinical presentation and the ongoing developmental changes characteristic of this age group (7). Therefore, the identification of objective and non-invasive biomarkers capable of supporting early diagnosis and patient stratification would contribute significantly to improving the understanding and treatment of schizophrenia and psychotic disorders.

At present, the pathophysiological mechanisms underlying psychosis are not fully defined (8). While the most widely accepted theory is that of the interaction between genes and environment, recent studies have highlighted the previously overlooked role of epigenetics (9, 10) in the development of schizophrenia and psychotic disorders.

Biological markers investigated during first-episode psychosis may offer a unique opportunity to explore molecular mechanisms associated with disease onset before the influence of long-term antipsychotic treatment, recurrent episodes, or chronic disease progression. In this context, peripheral biomarkers derived from blood samples have gained increasing interest, as they are minimally invasive, relatively inexpensive, and more accessible compared to central nervous system investigations (11, 12).

Micro-RNAs (miRNAs) are small, non-coding RNA molecules, approximately 18–25 nucleotides in length, that regulate gene expression at the post-transcriptional level. By binding to complementary sequences on target messenger RNAs (mRNAs), miRNAs can inhibit translation or promote mRNA degradation, consequently influencing a wide range of biological processes (13). In the central nervous system, miRNAs are involved in several biological pathways, including neurodevelopment, synaptic plasticity, inflammatory signaling, oxidative stress, and dopaminergic neurotransmission (14, 15). Circulating miRNAs can be isolated in tissues such as plasma, serum, whole blood, peripheral blood mononuclear cells, and extracellular vesicles, having the advantage of retaining high stability to enzyme degradation or pH variations (16, 17). Moreover, miRNAs are easily accessible when they are isolated from peripheral whole blood.

A systematic review identified the articles investigating the differently expressed miRNAs in psychotic disorders compared to healthy control groups (18). It is worth noting that most of them did not differentiate between age groups in the analysis, which may pose a problem, considering that miRNA expression has been proven to vary across developmental stages (19, 20). Moreover, chronicity may additionally contribute to neurobiological changes in the central nervous system (21).

Another factor that could significantly influence miRNA expression is treatment exposure; therefore, including treatment-naive subjects in the samples could yield clearer results (22).

Data regarding circulating miRNA profiles in Eastern European populations remain scarce, particularly concerning treatment-naïve patients with first-episode psychosis (FEP). In a previous explorative study, we analyzed 179 miRNAs in the plasma of 14 Romanian adolescents (7 FEP and 7 controls) and identified a signature composed of 21 miRNAs associated with first-episode psychosis (23). Given the limited replication of circulating miRNA biomarkers in psychotic disorders, the present study aims to replicate a panel of 10 candidate miRNAs selected from our previous exploratory signature based on high fold-regulation (FR) values and statistical significance, using an independent, enlarged cohort of 22 treatment-naïve FEP patients and 28 treatment-naïve healthy controls (CTRL).

## Materials and methods

A total of 50 Romanian adolescents and young adults (22 FEP and 28 CTRL) were enrolled in the study between March 2024 and June 2026, in accordance with the previously reported inclusion and exclusion criteria (23). The inclusion criteria for the FEP group were the following: i) A diagnosis of Acute and Transient Psychotic Disorder based on the International Statistical Classification of Diseases and Related Health Problems 10^th^ Revision (ICD-10). ii) No previous psychiatric diagnoses. iii) No history of psychotropic medication in the previous 3 months, or no more than 7 days of treatment in the current episode (Treatment-naïve). iv). Ages between 15–29 years. Participants were deemed ineligible for inclusion if they met any of the following exclusion criteria: i). Use of psychotropic medication within 7 days before blood collection or at any time within the preceding 3 months; ii). Prior diagnosis of any mental illness; iii) Any organic diagnoses that could cause psychotic symptoms, such as head trauma, epilepsy, encephalitis, or other diagnoses based on clinician assessment; iv) Any documented premorbid developmental abnormalities or intellectual disability; v) Substance use (including alcohol, illicit drugs); vi). The refusal of consent from the patient or guardian.

The present cohort was enrolled in the same hospital as the previous study, but did not include the patients and controls enrolled in the previous exploratory study. Informed consent was obtained directly from all adult participants (age> 18 years), all of whom were voluntarily admitted and possessed the capacity to consent, while written informed consent from the legal guardians of minors (ages 15–17) was obtained. The study was approved by the Prof. Dr. Alexandru Obregia” Clinical Hospital of Psychiatry ethics committee (No. 137/22.02.2024) and was conducted in accordance with the Declaration of Helsinki. The psychiatric assessment was performed as previously described using the Positive and Negative Syndrome Scale (PANSS) for psychosis, the Shortened Hamilton Depression Rating Scale (HDRS), also known as the Hamilton Depression Scale (HAM-D), to assess depressive symptoms, and manic symptoms were assessed using the Young Mania Rating Scale (YMRS) (23). All control participants were also psychiatrically screened by an experienced psychiatrist and had no current or lifetime Axis I psychiatric disorder; PANSS ratings in controls were collected only to confirm the absence of clinically relevant psychotic symptoms and thus should be interpreted as baseline anchor-point scores rather than clinically meaningful psychopathology. Moreover, all the controls were screened to ensure they had no history of chronic medical conditions and were free from any regular prescription at the moment of sample collection. **Table 1** summarizes the demographic and clinical data of the study cohort.

**Table 1:** Demographic and clinical characteristics of study participants.

Characteristic	FEP (n=22)	CTRL (n=28)	p-value
Age (Mean ± SD)	18.68 ± 4.05	19.59 ± 3.717	0.442^a^
Sex (%F)	59.09%	57.14%	0.890^b^
Smokers	31.8% current smokers 68.2% never smokers	10% current smokers 90% never smokers	0.085^b^
Alcohol consumers	95.45% non-drinker 4.55% occasional	100% non-drinker	0.254^b^
Coffee consumers	95.45% non-consumer 4.55% 1–3 cup/day	100% non-consumer	0.254^b^
Sleeping hours*	<5 hours (n=2) 5–6 hours (n=11) 6–7 hours (n=8)	<5 hours (n=0) 5–6 hours (n=1) 6–7 hours (n=27)	0.001^b^
PANSS Total (Mean ± SD)	91.18 ± 23.756	36.54 ± 6.692	<0.001^a^
PANSS Positive (Mean ± SD)	21.36 ± 4.865	9.21 ± 5.852	<0.001^a^
PANSS Negative (Mean ± SD)	21.91 ± 8.434	8.68 ± 2.709	<0.001^a^
PANSS General (Mean ± SD)	47.91 ± 12.980	18.64 ± 3.983	<0.001^a^
HDRS (Mean ± SD)	9.91 ± 4.230	3.14 ± 4.352	<0.001^a^
YMRS (Mean ± SD)	11.95 ± 6.572	2.64 ± 2.376	<0.001^a^

*Missing data for 1 FEP patient; ^a^Student’s t-test; ^b^Chi-squared test.

SD, standard deviation; PANSS Total, Positive and Negative Syndrome Scale Total Score (sum of all subscales, measuring overall symptom severity); PANSS Positive, Positive and Negative Syndrome Scale Positive Subscale (measures psychotic symptoms such as delusions and hallucinations); PANSS Negative, Positive and Negative Syndrome Scale Negative Subscale (measures deficits such as flat affect, emotional withdrawal, and apathy); PANSS General, Positive and Negative Syndrome Scale General Psychopathology Subscale (measures general psychiatric symptoms like anxiety, depression, and disorientation); HDRS, Hamilton Depression Rating Scale (measures the severity of depressive symptoms);YMRS, Young Mania Rating Scale (measures the severity of manic symptoms).

Procedures for blood collection, plasma separation, RNA isolation, and reverse transcription were performed as previously detailed (23). Plasma was frozen immediately after collection, stored -80 C, and subjected to no prior freeze-thaw cycles before RNA extraction. All processed samples were free of visible hemolysis. The levels of ten candidate miRNAs were analyzed using a miRCURY LNA SYBR Green PCR Kit and a miRCURY LNA miRNA PCR Assay (Qiagen): miR-23b-3p (YP02119314), miR-27a-3p (YP00206038), miR-30a-5p (YP00205695), miR-92b-3p (YP00204384), miR-125a-5p (YP00204339), miR197-3p (YP00204380), miR205-5p (YP00204487), miR-221-3p (YP00204532), miR-338-3p (YP00204719), and miR-1260a (YP00205892). All reactions were performed in technical duplicates, yielding reliable Ct values below 35 within the linear dynamic range, while clean No-Template Controls across both reverse transcription and qPCR stages confirmed the absence of reagent contamination. In the exploratory profiling study, miRNA expression was normalized using the global mean of the most expressed miRNAs on the array, which is not applicable in a targeted qRT-PCR validation of only 10 candidates. Therefore, in this replication study, we selected endogenous reference miRNAs using RefFinder, analyzing the Ct values of the 27 best-expressed miRNAs (Ct between 19 and 29) in our previous study (23). This initially identified let-7b-5p and miR-101-3p as the most stable. After qRT-PCR in the full cohort, miR-101-3p did not meet stability criteria and was excluded to avoid normalization bias; thus, normalization was performed using let-7b-5p (YP00204750) alone.

Because miRNA levels were non-normally distributed (Shapiro–Wilk test, p<0.05), the nonparametric Mann–Whitney test was used to assess differences between the FEP and CTRL groups, and fold change (FC) values were calculated to quantify relative expression differences between groups. Spearman correlations between miRNA levels and clinical data were computed. To account for multiple testing, p-values were corrected using the False Discovery Rate (FDR), and results were considered significant at FDR-adjusted p < 0.05. Additionally, the area under the receiver operating characteristic (ROC) curve (AUC) was calculated for significant miRNAs to evaluate their discriminative performance. Analyses were conducted in SPSS v17.0, and figures were generated with GraphPad Prism v8.4.3.

## Results

The FEP patient group and the CTRL group were comparable with respect to sex, age, alcohol consumption, and smoking status (p > 0.05). Although smoking showed a minor numerical difference, it did not reach statistical significance (p = 0.085), and the miRNA levels did not differ when comparing smokers (n = 10) versus non-smokers (n = 40) (p > 0.05 for all miRNAs). Regarding sleep duration, groups differed significantly, with FEP patients reporting fewer hours of sleep than controls (p = 0.001). To investigate whether sleep duration acted as a confounding factor, correlation analyses were performed between sleeping hours and candidate miRNA expression levels across cohorts. Because sleeping hours did not show statistically significant correlations with miRNA levels in the FEP group, CTRL group, or the entire cohort (p > 0.05), sleep duration was determined not to be a confounding variable for these biomarkers. Moreover, sleep disturbances and altered sleep duration are widely recognized as core intrinsic features of the psychopathology of first-episode psychosis rather than independent background characteristics (24).

Among the 10 selected miRNAs found showing different levels in FEP treatment-naïve patients in our previous exploratory study in a small cohort (23), miR-1260a was replicated in this study, which included 50 adolescents and young adults (22 FEP and 28 CTRL), considering as criteria for successful replication achieving statistical significance in the current independent cohort and demonstrating consistent direction and comparable magnitude of effect relative to the initial exploratory study. miR-1260a showed significant down-regulation in the FEP group compared to CTRL (raw p-value= 0.004, FDR Adj. p = 0.04, FC = 0.53) (**Figure 1A**) alongside a moderate discriminatory performance (AUC = 0.741, p = 0.004) (**Figure 1B**). Furthermore, correlation analyses conducted exclusively within the FEP patient group indicated a negative correlation between miR-1260a levels and the positive PANSS score (p = 0.017, rho = -0.503), which did not retain statistical significance after FDR correction (FDR adj. p = 0.102) (**Figure 1C**).

**Figure 1:**
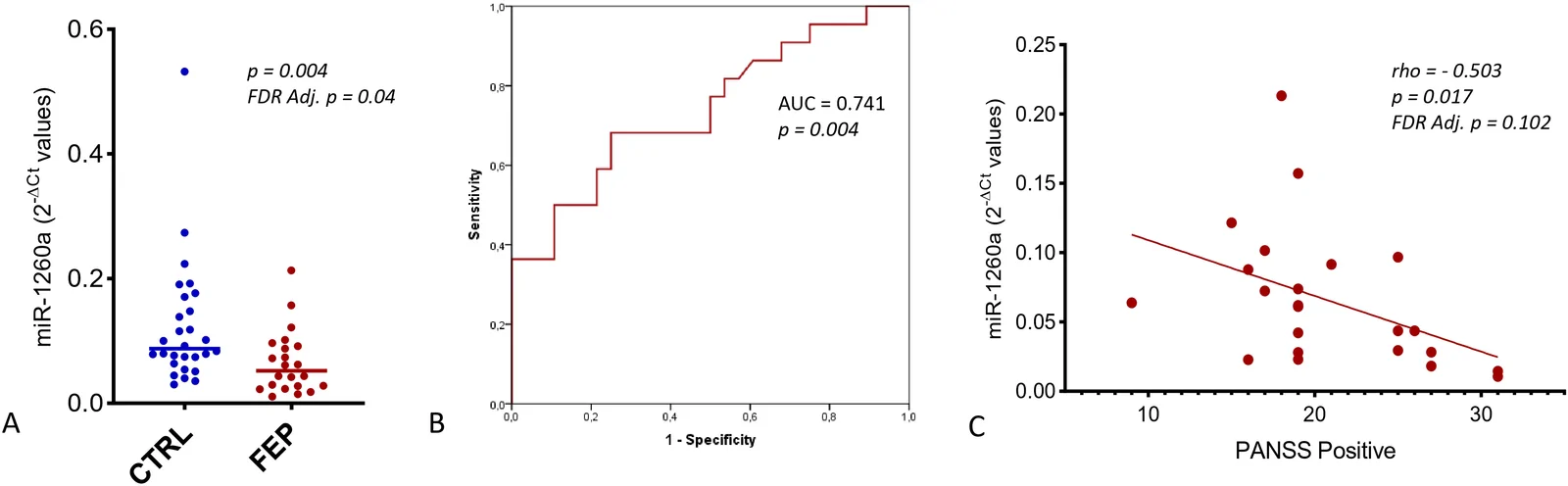
Level, discriminative performance, and clinical correlation of miR-1260a in FEP and CTRL groups. **(A)** Individual 2^-ΔCt^ values of miR-1260a for each subject shown as colored dots (CTRL in blue, FEP in red). The horizontal bar within each group represents the median miRNA level. Statistical significance between groups was determined using the Mann–Whitney U test followed by FDR correction. **(B)** ROC curve demonstrating the moderate discriminative performance of miR-1260a between FEP and CTRL groups. **(C)** Correlation analysis within the FEP patient group indicating a negative correlation between miR-1260a plasma levels and the positive PANSS (Positive Scale component of the Positive and Negative Syndrome Scale).

## Discussion

Building upon our previous exploratory findings, the present study successfully replicated miR-1260a from a panel of ten candidate circulating miRNAs in a larger cohort of treatment-naïve Romanian adolescents and young adults diagnosed with FEP. Our results demonstrate significant downregulation of miR-1260a compared with age- and sex-matched healthy controls, together with moderate discriminatory performance, suggesting that this molecular alteration is present at illness onset. Independent replication is a key step toward biomarker development in psychosis, where cross-study reproducibility has historically been limited for peripheral epigenetic markers, including miRNAs (25).

The downregulation of miR-1260a observed here is consistent with the direction reported in our exploratory study (23). Beyond psychotic disorders, altered expression of miR-1260a has previously been reported in patients with depression and anxiety associated with inflammatory bowel disease (26), as well as in Alzheimer’s disease (27), although it remains uncharacterized in psychiatric disorders. The latter finding further suggests that alterations in miR-1260a expression may occur across different psychiatric and neurological conditions. However, findings from other diagnostic groups should not be interpreted as evidence that the same downstream targets or mechanisms are operating in FEP, because miRNA effects are often highly context-dependent (cell type, developmental stage, tissue source, comorbidities, and experimental conditions), and directionality can differ across cohorts and disorders. Accordingly, in the absence of mechanistic experiments in psychosis-relevant tissue or models, the present results support miR-1260a primarily as a replicated peripheral association with FEP rather than a defined causal pathway.

An exploratory finding of our study was the negative correlation between circulating miR-1260a levels and PANSS positive symptom severity scores within the FEP group, although this association did not retain statistical significance after FDR correction. This pattern is compatible with the possibility that some peripheral miRNA alterations may relate not only to case–control status but also to symptom dimensions; however, given the modest sample size and multiple-testing burden, this observation should be interpreted cautiously until replicated. Importantly, the current cross-sectional design does not allow causal inference, and peripheral circulating miRNAs should be interpreted as peripheral molecular correlates rather than direct proxies of central nervous system molecular activity (28).

The observation that miRNAs can be dysregulated across multiple psychiatric phenotypes may reflect biological overlap across conventional diagnostic categories. Genetic and regulatory studies indicate that miRNA-related regulatory networks are relevant to psychiatric risk architecture, and may be shared across diagnostic categories, which may reduce the specificity of any single miRNA as a disorder-specific biomarker (29, 30). From this perspective, circulating miRNAs may ultimately prove most useful when integrated into multimodal models, such as molecular panels combined with clinical and other biological measures, rather than being interpreted as standalone mechanistic indicators for FEP.

Although altered circulating miRNA profiles have been reported in schizophrenia and related disorders, relatively few studies have focused specifically on treatment-naïve first-episode samples, and even fewer have examined adolescents and young adults. Limited reproducibility in the field likely reflects heterogeneity in sampling, analytical pipelines, ethnicity, illness stage, substance exposure, and particularly medication status (18, 28). By replicating miR-1260a using the same methodology as our previous study in an independent cohort, the current study addresses a key limitation of the literature, namely, the scarcity of independent validation.

One of the major strengths of the present study is the inclusion of treatment-naïve Romanian patients diagnosed with FEP. Antipsychotic medication has been shown to influence peripheral miRNA expression, making it difficult to distinguish disease-associated molecular alterations from treatment-related effects in studies including chronically treated patients (22). By investigating individuals before prolonged pharmacological exposure and during the early stages of illness, the present findings are more likely to identify biomarkers associated with psychosis itself rather than secondary changes resulting from medication or chronic disease progression. In addition, restricting the sample to adolescents and young adults could minimize the confounding effects of aging, as circulating miRNA expression is known to vary across developmental stages. Taken together, the combination of treatment-naïve status, independent replication, and a relatively homogeneous developmental age range reduces several major sources of biological heterogeneity that have limited previous circulating miRNA studies.

To our knowledge, this is the first independent validation study to investigate circulating miRNA expression in treatment-naïve Romanian adolescents and young adults with FEP. This expands the available evidence beyond the populations most frequently studied and contributes to a more geographically diverse assessment of candidate biomarkers. Because miRNA profiles can be shaped by genetic background and environmental exposures, validation across populations is important for establishing generalizability and reproducibility.

This study has several limitations. First, although larger than our previous pilot study, the sample size remains relatively modest and limits power for smaller effects and clinical correlations. Second, the cross-sectional design does not allow for the evaluation of temporal changes in miRNA expression following treatment or during disease progression. Third, circulating plasma miRNAs originate from multiple peripheral tissues and cannot be assumed to reflect brain-specific mechanisms. Moreover, BMI, which could represent a possible confounding factor, was not available for all the participants. Finally, although candidate miRNAs were selected based on our previous exploratory findings, functional validation and mechanistic studies were beyond the scope of the present work and will be necessary before drawing conclusions about biological pathways in FEP.

## Conclusions

In conclusion, treatment-naïve Romanian adolescents and young adults with FEP exhibit a distinct circulating plasma miRNA profile characterized by replicated downregulation of miR-1260a, which shows moderate discriminative performance and a preliminary association with positive symptom severity. These findings support miR-1260a as a candidate peripheral biomarker signal present near illness onset; however, mechanistic interpretations based on miRNA studies from other disorders should be considered contextual and hypothesis-generating rather than evidence of shared mechanisms in FEP, particularly given the significant clinical and molecular overlap with other psychiatric phenotypes that share similar symptom dimensions. Larger longitudinal studies integrating transcriptomic, clinical, and neuroimaging data will be necessary to determine their diagnostic and prognostic utility.

## Data Availability

The raw data supporting the conclusions of this article will be made available by the authors, without undue reservation.
